# Editorial: The Role of Play in Child Assessment and Intervention

**DOI:** 10.3389/fpsyg.2017.01098

**Published:** 2017-06-29

**Authors:** Silvia Salcuni, Claudia Mazzeschi, Claudia Capella

**Affiliations:** ^1^Department of Developmental and Socialization Psychology, University of PaduaPadua, Italy; ^2^Dipartimento di Filosofia, Scienze Sociali, Umane e Della Formazione, University of PerugiaPerugia, Italy; ^3^Department of Psychology, Universidad de ChileSantiago, Chile

**Keywords:** play, assessment, intervention, affect, cognition, mother-child interaction, attachment

In early 2016 we decided to open a Topic about children play, to highlight its important role in child psychological assessment and intervention. Since early 1900's in psychological literature it has been raised the multiple functions of play in child development and the use of play in assessment and treatment with children (Gil, 1991). Play provides children with valuable cognitive, emotional, and interpersonal learning opportunities. The significance of play in childhood has led to its frequent use in the assessment of child development and in the implementation of child and parent-child psychological and educational interventions. Historically, few studies provided a rigorous validation of play assessment measures and empirical evaluation of intervention or psychotherapy efficacy; just a few works have included parents, dyads, or atypical child development. So, as a paradox, even play is a ubiquitous and universal aspect of early childhood, worldwide recognized in its importance for children development, it was not so easy to find a group of colleagues that rigorously evaluated validity and efficacy of assessment and interventions based on children play. We are pleased to present to the readers a final research E-book that includes the work of 60 authors from many states of America, Austria, Brazil, Chile, India, Italy, Turkey, and UK, and showed the most current literature on the use of play in child assessment and intervention in its 15 articles.

Among those articles, various study the role of play in assessment. Within those, the state of the art about psychometric and cognitive characteristics of most common play and coping measurement instruments was proposed by Capurso and Ragni. They reviewed and compared the principal developmental functions of play with the different stages of the coping process, proposing that play should be considered an elective form of coping with most aspects of children's lives, highlighting the necessity to adopt methodologies that toward play enable an accurate recognition of coping. To fill the gap about the paucity of standardized and valid measures specifically devoted to assess the core domains involved in play activities, a series of papers were devoted to measure external and construct validity of the Affect in Play Scale-Preschool (4–5 years APS-P) and its Extended Version (6–10 years APS-P Extended Version), semi-structured parallel tools designed to explore child's cognitive and affective processes using a 5 min standardized play task. Delvecchio et al. validate APS-P and its Extended Version to 538 Italian children aged 4–10, assessing gender as well as age differences. Results supported the use of both tools as adequate measures to assess the interplay of cognitive and affective skills in preschool and school age children. These tools exist also in Brief version (APS-P-BR and APS-P-BR Extended Version) scored *in vivo*, without video-recording procedures. In wide sample Italian children aged 4–10 years, Di Riso et al. showed how APS-P-BR and its Extended Version factor scores were strongly related to APS-P Extended Version factor scores suggesting that the APS-P-BR and its Extended Version are an encouraging brief measure to assess pretended play using toys. Through the use of APS, Federici et al. investigated 63 scholar children's representations of the three main models of disability (medical, social, and biopsychosocial) and how these models affected cognitive and affective components of non-clinical children's play. Results showed significant effects on the affective components of play, and no-one on cognitive components or on a variety of affect, demonstrating that, when children are involved in pretend play, independently from which concepts of disability emerge, they exclusively related to the medical model of disability. George and Solomon examined the predictive validity of the Attachment Doll Play Assessment to caregiving accessibility and responsiveness assessed from mother-child interaction and maternal representation. Sixty-nine mothers and their 5–7-year-old children were observed during a pre-separation dyadic interaction task. Child security with the mother was associated with positive dyadic interaction and flexibly integrated maternal caregiving representations. Child controlling/disorganized attachments were significantly associated with problematic dyadic interaction and dysregulated-helpless maternal caregiving representations. Standing within the dyadic interaction, Fadda and Lucarelli investigated longitudinally the relations between mother–child interactions during feeding and play and child's pre-verbal communicative abilities in extra-dyadic interactions during play, in 20 dyads comparing those with functional interactions vs. dysfunctional interactions. A stable relation over time between mother–child interactions and child's social communicative skills in extra-dyadic interactions emerged. Another important longitudinal design was used by Salvatori et al., highlighting the preterm birth weight as a strong risk factor for early mother-infant interactions. Ten minutes of mother-child play interaction were recorded and later coded according to the Emotional Availability Scales. Preterm birth weight affects the quality of mother-toddler interactions, especially in the case of Extremely Low Birth Weight children.

Remaining within the field of play assessment, three papers put their attention to samples of children with clear disabilities. Bentenuto et al. analyzed 75 mothers' involvement in child play sessions, using a coding system for exploratory and symbolic play. Children presented Autism Spectrum Disorder, Down Syndrome, and typically developing children. Results indicated that children with ASD showed more exploratory play compared to children in the other groups and no significant differences emerged between the three groups for child symbolic play or for mother play. Using rigorous observational methods to assess spontaneous pretend play, Kang et al. analyzed predictors and moderators of spontaneous pretended play in children with and without Autism Spectrum Disorder. Results showed the negative effect of ASD symptoms on pretend play was simultaneously moderated by low ToM and high verbal ability, both related to less pretend play production among children with more ASD symptoms. In a sample of 40 children affected by Down Syndrome, Fasulo et al. explored the video records of an interaction between a child and a practitioner, during the administration of the Bayley Scale of Infant and Toddler Development, using Conversation Analysis approach. The analysis of the sequences shows that the assessor promotes the child's engagement by coaching the actions required to administer the item in utterances with marked child-directed features. Moreover, the objects constituting the test item did not suggest to the child a unique course of action, leading to the assessor's modeling of the successful sequence. Authors proposed different important argumentation about data.

Even though there is substantial evidence that play-based therapies produce significant change (Ray et al., 2001), the specific play processes in treatment remain quite unexamined. A longitudinal qualitative study in the psychotherapeutic process of children who have been sexually abused was carried out by Tornero and Capella. Participants between the 7 and 10 years old were observed during three sessions of sand tray at different moments of therapy. Results revealed common and transversal forms of playful expression among this group of children shown by their engagement with sand-play, from the elaboration of personal stories that feature violence as a central theme, passing through the expression of their need for care and protection. The shifting dynamics of sandplay at each stage of therapeutic treatment is an important finding of this study that reveals the progress made during psychotherapy. Halfon et al. measured processes of change in long-term psychodynamic play: the Children's Play Therapy Instrument was used generating discrete and measurable units of play activity arranged along a continuum of four play profiles; through non-linear models, the results showed a picture of how these children express different psychic states in play, captured through the lens of play profiles, and begin to modify less dysfunctional profiles over the course of treatment. Finally, Salcuni et al. focused on a single case process-outcome design to evaluate play and verbal discourse of 30 psychotherapy sessions of a 3-year once-a-week psychodynamic psychotherapy, carried out with a 3-year-old girl. The Play Category System, the APS-P and Verbal Production, empirically measured, showed significant changes along the unfolding of the therapy: verbal production and discourse ability progressively increased and took the place of play, which instead became more symbolic.

In the main frame of psychotherapy, very impressive is the opinion by Ramires. Passing through a psychoanalytical as well as attachment theory review, Ramires focused on psychotherapeutic process, demonstrating that playing is an essentially intersubjective activity, that enables the construction/organization of internal and external realities. In line with this viewpoint, Rice, with a commentary about Shaheen's paper (2014) proposes that psychoanalytic psychotherapy with children advances executive function development through its promotion of implicit emotion regulation capacities through the technique of defense analysis, using programs based on common children games. Guidelines about how to develop play abilities either within the family, the nursery and pre-school context and, of course, the clinical setting, are proposed.

This collection of articles shows the development of several research with different population and research techniques. However, as a whole, contribute to the evidence of the important role of play in psychological assessment and treatment in children.

## Author contributions

All authors listed have made a substantial, direct and intellectual contribution to the work, and approved it for publication.

## Conflict of interest statement

The authors declare that the research was conducted in the absence of any commercial or financial relationships that could be construed as a potential conflict of interest.
